# Critical transitions in suspended sediment dynamics in a temperate meso-tidal estuary

**DOI:** 10.1038/s41598-019-48978-5

**Published:** 2019-09-04

**Authors:** T. J. S. Cox, T. Maris, T. Van Engeland, K. Soetaert, P. Meire

**Affiliations:** 1 Ecosystem Management Research Group, University of Antwerp. Universiteitsplein 1C, 2610 Wilrijk, Belgium; 2 Estuarine and Delta Systems, Netherlands Institute of Sea Research (NIOZ), P.O. Box 140, 4400 AC Yerseke, The Netherlands

**Keywords:** Element cycles, Environmental impact, Physical oceanography

## Abstract

There is growing consensus that human interventions can fundamentally change fine sediment transport in estuaries. Critical transitions in response to human interventions have been hypothesized based on indirect observational evidence and theoretical understanding. So far direct evidence has been lacking. Based on a 20 year data-set of surface suspended particulate matter (SPM) concentrations, we present empirical evidence of critical transitions in a temperate meso-tidal estuary. In 2008–2009 the SPM dynamics of the Scheldt estuary (Belgium/The Netherlands) changed dramatically. Not only did the total amount of sediment in suspension increase, a new maximum turbidity zone (MTZ) at typical winter discharges appeared. At intermediate and low summer discharges the longitudinal distribution of SPM now flickers between two markedly different states. Our data suggest that a range of human interventions (fairway widening and deepening, dredging and dumping activities) set the scene leading to the observed transitions. Moreover the freshwater MTZ in the Scheldt and in its major tributary exhibit an increasing sensitivity towards freshwater discharge, coinciding with water quality improvements. This suggests large scale impacts of changes in eutrophication status on estuarine sediment dynamics. This has largely been a blind spot in morphodynamic research.

## Introduction

One of the key defining characteristics of estuaries is their ability to effectively trap marine and fluvial sediments^1^. In many estuaries human interventions have altered the trapping of sediments. This has often lead to marked changes in suspended particulate matter (SPM) concentrations. Either the influx of sediments from the watershed was changed through the construction of dams and weirs (e.g.^2–4^), the retention of sediments and the exchange with the coastal zone was altered by changes in hydraulic regime or by morphological interventions (deepening, widening, embankments, the building of harbour infrastructure) (e.g.^5-9^), or dredging and dumping activities directly impacted SPM concentrations (e.g.^6,10^). Often, a combination of factors are at play. Over the past years the research community has taken renewed interest in estuarine suspended particulate matter (SPM) dynamics. This has been triggered by dramatically changed SPM dynamics resulting in hyper-turbid conditions in certain heavily impacted estuaries^11,12^. These changes are commonly attributed to engineering works that took place over the past decades^12^. It is hypothesized that these interventions affected the estuarine geometry to such extent that a seemingly unbounded upstream pumping of SPM led to extremely high concentrations and the formation of thick layers of fluid mud^13,14^. But also without the dramatic transition to hyper-turbid conditions, it has been postulated that fairway deepening can result in the appearance of a secondary maximum turbidity zone well beyond the classical location near the end of the salinity intrusion^15^. However these hypotheses are supported by indirect observational evidence: limited data availability only allows for a partial quantification of historic and current SPM distribution patterns. Discerning gradual from critical transitions and pinpointing the exact timing of hypothesized shifts is often impossible. So far direct evidence of critical transitions in estuarine sediment transport and distribution patterns have been lacking.

While observing changes in SPM is already a challenge, explaining those changes is even more difficult because of the intrinsic complexity of fine sediment transport. Indeed the balance between upstream and downstream forces that govern particulate transport is delicate. Downstream forces are primarily the result of advection driven by freshwater inflow. But also gravitational forces along the bottom slope and longitudinal density gradients can be important^16^. Upstream, counter-gradient forces are predominantly the result of estuarine circulation and asymmetry in tidal velocities and in flood and ebb duration^1^. However, also asymmetry in flocculation dynamics^17^, in horizontal and vertical turbulent mixing^16^ can contribute to net upstream transport. At high SPM concentrations, vertical density gradients can result in reduced vertical mixing^18^ potentially affecting the flow and introducing further asymmetry and upstream fine sediment transport^19^. While the longitudinal sediment transport balance is delicate, the relative importance of processes governing the vertical balance is fundamentally different when at low or high concentrations. Hindered settling, only important at high concentrations, is a crucial process to understand shifts towards hyper-turbid estuaries^20^.

Sediment properties are strongly influenced by biochemical and biological processes. Organic matter content, biofilms by bacteria and microphytobenthos and the disturbance by macrobenthic organisms are known for their importance in determining the erodability of fine sediments^21^. Also the aggregation-disaggregation dynamics and associated settling velocities of aggregates are affected by biological activity. The biophysical determinants of aggregation-disaggregation dynamics of cohesive sediments is currently a hot research topic (e.g.^22^). In estuaries this is particularly important since the settling velocity of aggregates co-determines the location and intensity of the maximum turbidity zone. Theoretical results show that increasing settling velocities can result in an upstream shift of estuarine turbidity maxima^15,23^. Thus, there is potentially a strong link between the changes in eutrophication status and the large scale distribution of fine sediments in estuaries. This is, however, largely a blind spot in current research.

**Figure 1:**
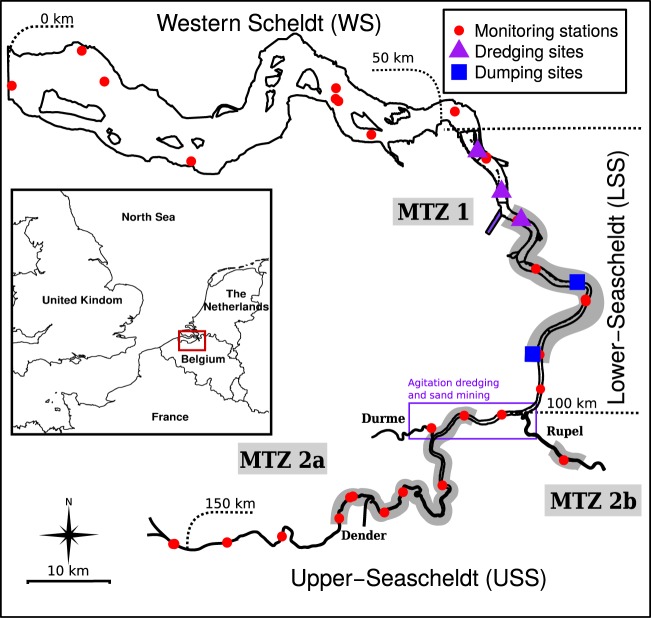
The Scheldt estuary. Monitoring stations, important dredging and disposal sites and maximum turbidity zones in the Scheldt estuary.

Consistent long-term estuarine data-sets of SPM and relevant environmental parameters are sparse. With a more than 20 year monitoring record covering physics, biochemistry and biology, the Scheldt is one of the most intensely monitored estuaries in the world. Situated in Northern Belgium and the Southwest of The Netherlands (Fig. 1), the tidal wave enters deeply inland where it is blocked by weirs resulting in 240 km of estuarine tidal reaches. The Scheldt is one of the most impacted estuaries in Europe in terms of organic and chemical pollution^24–26^. Also its morphology has been strongly altered^14^, and large volumes of sand and mud are continuously dredged for fairway maintenance and to guarantee ship accessibility to docks^27^. The Scheldt has 2 major branches, the Rupel and the upper Sea Scheldt (USS, Fig. 1), with similar freshwater discharges. Water treatment efforts led to rapid changes in water quality, first in the USS (around 2003^26^), and later in the Rupel tributary^28,29^.

In his paper we analyze a unique 20-year data-set of surface SPM. Bearing in mind the morphologic interventions and changes in eutrophication status during this period, we search for the existence of shifts in longitudinal SPM distribution patterns, and whether these occur as gradual changes or as critical transitions. We look for interactions of sediment dynamics with the human-induced improvements in water quality, testing the hypothesis that changes in eutrophication status can have system scale impact on estuarine sediment distribution.

## Results

### Spatio-temporal patterns in surface SPM

**Figure 2:**
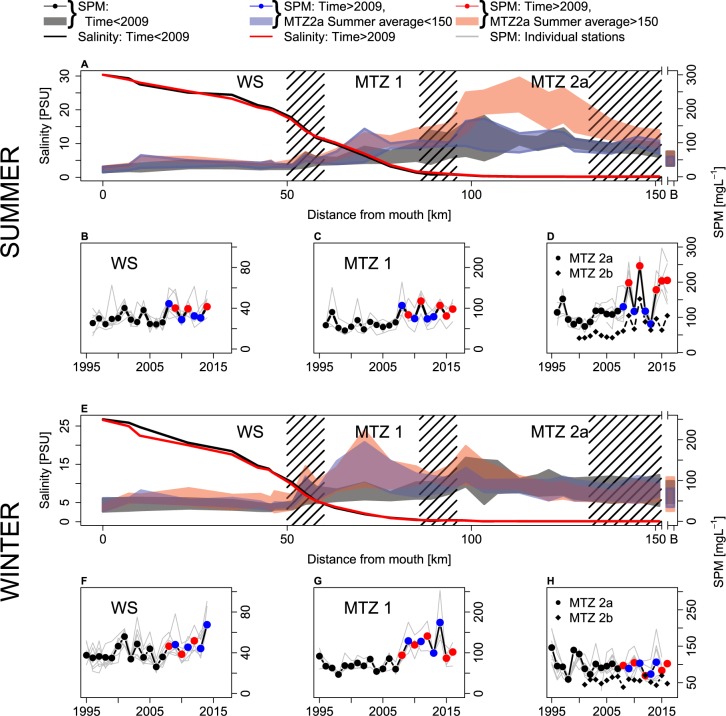
Spatio-temporal patterns in SPM. Longitudinal distribution of Salinity and SPM in summer (**A**) and winter (**E**). White bands indicate the characteristic zones of the estuary with regard to SPM dynamics. Hatched out areas represent transitions between characteristic zones. “B” on x-axis indicates concentrations at boundary (upstream of the weir). Summer (**B**–**D**) and winter (**F**–**H**) averaged time series of SPM in characteristic zones (note the different scale on y-axes). Light grey lines represent time series at all individual monitoring stations within each zone; black lines and black, blue and red dots represent zone-averaged time series.

The Scheldt estuary can be subdivided in 4 turbidity zones, based on the seasonally averaged concentrations at the sampling locations (Fig. 2B–D,F–H). Lowest concentrations are found in the Westerschelde (WS, 0 km–50 km from the mouth; 1995–2015 mean: 36.9; sd: 17.2). A first maximum turbidity zone is found between 60 and 80 km from the mouth (MTZ 1; 1995–2015 mean: 81; sd: 47.5). Largest concentrations are found in a 30 km stretch in the major tributary to the estuary, the upper Sea Scheldt (MTZ 2a, 100 km–130 km from the mouth; 1995–2015 mean: 112.6; sd: 56.7). Concentrations in the Rupel (MTZ 2b) are lower than in the USS (mean: 62; sd: 37.5), but inter-annual variability of Summer concentrations is similar as in MTZ 2a.

The time series of seasonally averaged concentrations in the 4 zones show clear trends. Until 2009, annually averaged concentrations in MTZ 2a were about 100 mg L^−1^. Starting from 2009, the summer averaged concentrations exhibit large variation with more than a factor 3 difference between lowest (81 mg L^−1^, 2013) and highest (247 mg L^−1^, 2011) concentration (Fig. 2D). Such fluctuations can also be observed in the second tributary MTZ2b, although at lower concentrations. Summer averaged concentrations in MTZ1 show an increasing trend over 1995–2015 (slope = 2 mg L^−1^ yr^−1^, p < 0.001, R^2^ = 0.566, Fig. 2C). In contrast, winter averaged concentrations in MTZ1 show a marked increase around 2008 (Fig. 2G): before then, average winter concentrations were rather constant at 71mg L^−1^. After 2008, winter concentrations show more variability and on average almost doubled to 125 mg L^−1^. In the most downstream part of the estuary, 0–50 km, annual average concentrations show a weakly significant increase, at a rate of 0.5 mg L^−1^ yr^−1^ (p < 0.05, R^2^ = 0.21) (Fig. 2F). Winter concentrations in MTZ 2a and MTZ 2b did not change noteworthy in the investigated period (Fig. 2H). The observed changes in SPM are contrasted by virtually no changes in seasonally averaged longitudinal salinity profiles.

The Summer longitudinal SPM distribution exhibit a remarkable feature: certain years after 2008 show increased summer concentrations in MTZ 2a when compared to the period before, while concentrations in others years are not different from the earlier period (Fig. 2A,D). These increased summer concentrations are not caused by increased inputs from the catchment: boundary concentrations upstream the weir are lower than concentrations in the estuary and show no trend (Fig. 2A).

### Timing and character of change

**Figure 3:**
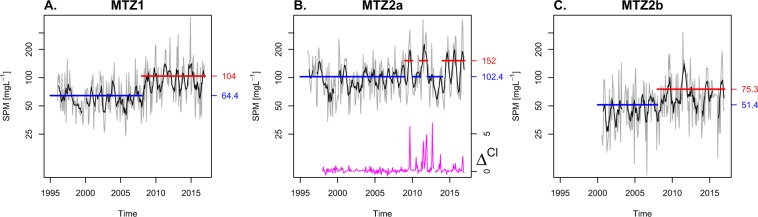
Changepoints in SPM time series. Time series of monthly SPM concentrations (grey lines) and 6 month moving average (black lines) in the maximum turbidity zones. Horizontal coloured lines are averages of monthly concentrations, before and after detected changepoints. The magenta line denotes the difference between Chloride concentration at 121 km and upstream boundary of the estuary.

Change point analysis (see Methods) detected a shift in the mean of the monthly SPM time series in MTZ1 and MTZ2b in early 2008 (Fig. 3A,C). In MTZ1 the average concentration increased from 64.4 to 104 mg L^−1^; In MTZ2b from 51.4 to 75.3 mg L^−1^. The pattern of change is more delicate in MTZ2a; periods with and without elevated concentrations are found since 2009 (Figs 2, 3), hence no change point is automatically detected. Nevertheless, a significant difference in mean concentration is found between years with high concentration (152 mg L^−1^) and the years with low concentrations grouped with years prior to 2009 (102.4 mg L^−1^). The first year with elevated SPM concentrations in MTZ2a is 2009. Chloride data provides more evidence for 2009 as the year of change. Until 2009, the Chloride concentrations at 121 km from the mouth are virtually the same as concentrations of the water entering the estuary at the upstream boundary – the difference (Δ^Cl^) fluctuates around zero (Fig. 3B). From 2009, the concentration difference shows marked spikes indicating a deeper salt intrusion.

### Alternative co-existing responses to FW discharge and long term change in relation to water quality improvement

**Figure 4:**
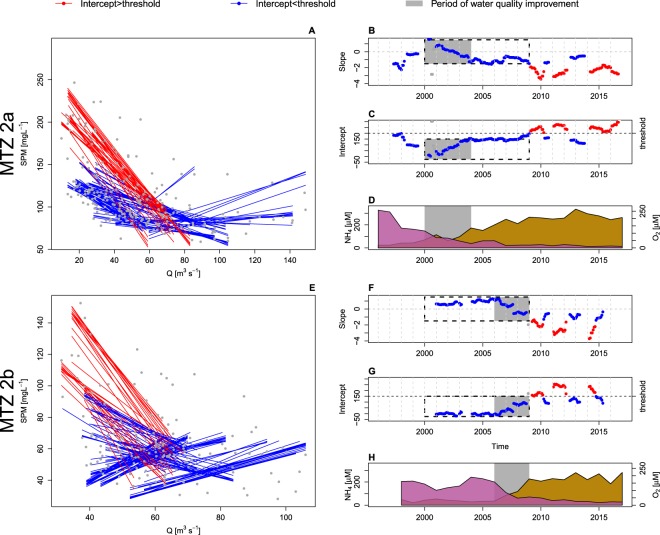
Changing sensitivity of SPM to freshwater discharge. Relation between 6 month averaged SPM concentration in MTZ2a and MTZ2b and 6 month averaged freshwater discharge (**A**,**E**). Coefficient of the regression lines in moving windows of 12 months (**B**,**C**,**F**,**G**). Annually averaged ammonium (NH_4_) and oxygen (O_2_) concentrations (**D**,**H**). Grey bands corresponds to periods of water quality improvement.

The large variability in monthly averaged concentrations notwithstanding, the 6 month moving averaged concentrations show a clear quasi-periodic behavior (Fig. 3). In MTZ2a and MTZ2b this is strongly linked to freshwater inflow into the respective tributaries. Figure 4A,E show the correlation between 6 month moving averages of SPM and Q. Superimposed are line segments representing lagged linear model fits in moving windows of 1 year width (see Methods).

The evolution of the regression coefficients (Fig. 4B,C,F,G) show the appearance in 2009 of regressions with higher intercepts and steeper slopes. While before 2009 the intercept in MTZ2a and MTZ2b was below 200 mg L^−1^, starting from 2009 the intercept switches between periods with a high intercept (~250 mg L^−1^) and a low intercept (~150 mg L^−1^). As the intercept is the extrapolated SPM concentration at zero discharge, this could loosely be interpreted as the maximal amount of SPM that can possibly be found in the respective MTZs during summer conditions. At the same time the slopes switch between steep (~−2.5 mg L^−1^/(m^3^ s^−1^)) and less steep (−1 mg L^−1^/(m^3^ s^−1^)). The slope indicates the sensitivity of SPM in the MTZs for changes in upstream discharge. Thus, starting from 2009 two co-existing ‘equilibrium’ SPM concentrations co-exist at similar freshwater discharges. During certain periods, a high amount of SPM is found at low freshwater discharge, while in other periods it is not.

The regression coefficients exhibit a remarkably consistent trend during periods of water quality improvement. With increasing oxygenation of the water column and decrease in dissolved ammonium concentrations (Fig. 4D,H), the intercepts steadily increase (from ~0 to ~150 mg L^−1^) while slopes change from positive to negative and gradually become steeper (from ~1 to ~−1.5 mg L^−1^/(m^3^ s^−1^)). The changes in water quality happened first in MTZ2a (around 2003), and later in MTZ2b (around 2007) as a result of a different timing in water treatment efforts^26,28,29^. In MTZ2b, the period preceding water quality improvements is characterized by positive slopes and zero intercepts, indicating that SPM concentrations in this period were governed by input from the catchment. Maximal erosion in the Belgian loam belt, draining into the Rupel, occurs well before maximal winter discharges, and fine sediments are mainly exported in early winter^30,31^ with maximal import rate of fine sediments from the catchment occurring before maximal discharges, and best regressions have negative lag (see Methods). After water quality improvement MTZ2b behaves similar to MTZ2a: negative slopes, negative intercepts, and positive lags then indicate that SPM is governed by upward transport.

## Discussion

In their review of mechanisms governing estuarine MTZ formation, Burchard *et al*. categorize MTZs according to 3 different phenomenological types with different associated transport mechanisms: trapping at the salt intrusion limit, trapping in the freshwater zone and topographic trapping^1^. The location of the post 2008/2009 MTZs are in accordance with this phenomenology: MTZ1 sits at the salt intrusion limit, MTZ2a and MTZ2b in the freshwater zone. Since the summer and winter MTZs are of different phenomenological type, the seasonal shift between both suggests a shift in dominant upstream transport mechanism. Such dynamics has also been observed in the Gironde estuary: exchange flow is the dominant upstream transport mechanism during high freshwater discharges and tidal asymmetry during low discharges^32^. Such shift in dominance does not need to be estuary-wide, but only at the end of salinity intrusion during summer discharges where one would expect the MTZ to sit if exchange flow would be the dominant upstream transport mechanism (roughly between 80 km and 100 km, Fig. 2a). This offers a plausible explanation for the MTZ dynamics in years with high summer SPM in the USS, but does not explain the flickering between alternative longitudinal sediment distribution (Fig. 2). Moreover, one of the major advances over the last few years is precisely that MTZ formation is the result of a delicate balance of transport phenomena. As Burchard *et al*. Noted: “[Various] trapping mechanisms usually work simultaneously, and assessing which mechanism is essential for trapping in a specific estuary requires a detailed analysis of the SPM transport contributions.” Unraveling this balance is not possible based on our data-set alone. To precisely identify which transport mechanism lead to the observed MTZ dynamics in the Scheldt, we need additional data-analysis (e.g. of tidal asymmetries), perhaps additional field measurements and dedicated modelling, all of which are beyond the scope of the current paper.

In the following we discuss the possible causes of the described transition in 2008/2009. The diverse processes that affect SPM dynamics (physical, biogeochemical and biological) and the human interventions that took place simultaneously, impede straightforward explanation of the observed changes. Nevertheless, we can draw some conclusions based on the timing of events.

### Impact of fairway deepening and widening on salinity intrusion and MTZ formation

**Figure 5:**
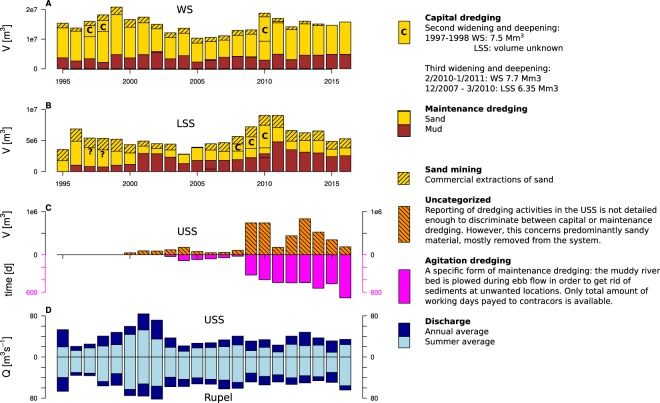
Overview of main morphologic interventions and freshwater inputs. Dredging and sand mining activities in Western Scheldt (**A**), lower Sea Scheldt (**B**), and upper Sea Scheldt. Summer and annual average of freshwater discharge in MTZ2a and MTZ2b (**D**).

At a given freshwater discharge, estuarine cross-section is a prime determinant of longitudinal turbulent transport of salt in vertically well mixed estuaries^33^. Therefore, the deeper salt intrusion witnessed by the Chloride peaks since 2009 (Fig. 3B) likely results from widening and/or deepening of the channel, probably triggered by dredging and sand mining activities that took place since 2009 (Fig. 5C). The fact that high summer SPM concentrations in MTZ2a simultaneously start to occur, suggests that the same intervention triggered the 2009 shift in SPM in MTZ2a. The increased artificial resuspension due to agitation dredging, which also increased since 2009, could have further increased SPM concentrations.

The inference that at a given flushing rate an increase in estuarine cross-section enhances retention, may be intuitive but processes that determine estuarine SPM dynamics are all affected differently by deepening and widening. For instance tidal asymmetry is a powerful transport mechanism for suspended particles^1^, and the impact of widening and deepening on tidal asymmetry can both promote or impede upstream transport. For the Ems estuary, theoretical and modelling studies have shown that deepening makes it more difficult to flush sediment out of the system^15^, and the idea that deepening has caused enhanced up-estuary transport of SPM in the Ems is now accepted^20^. To date, such analysis has not been performed for the Scheldt estuary.

Nevertheless, not only changes in MTZ2a in 2009 coincide with local widening and deepening, also the increase in SPM concentrations in MTZ1 (Fig. 3A) in 2008 coincide with the start of capital dredging works in the downstream parts of the estuary (Fig. 5B). To deepen and widen the fairway towards the harbor of Antwerp a total amount of 6.35 10^6^ m^3^ of sand was dredged and mostly disposed back in the estuary. Deepening and widening in the outer part of the estuary (Westerschelde) took place later (2010–2011); there about 7.7 10^6^ m^3^ of sand was dredged and disposed back in the estuary^34^. In contrast, sand mining activities and dredged volumes of sand for maintenance show no particular trend or changes (Fig. 5A,B; for a discussion of dredging and dumping of mud, see below). These observations support the hypothesis that deepening and widening is a likely cause for the abrupt changes in 2008 and 2009 in SPM dynamics. The appearance of a second MTZ after fairway deepening has indeed been hypothesized before^13,15^. However, we should also consider other potential causess.

### The dredging pump

Over the last two decades, the amount of artificially relocated mud by dredging and dumping in the LSS has been increasing. Total artificial fine sediment transport averaged 0.7 10^6^ m^3^ yr^−1^ in 1996–2000 and increased to 3.3 10^6^ m^3^ yr^−1^ in 2010–2015, with the largest volume (4.7 10^6^ m^3^ yr^−1^) relocated in 2011. These rates are an order of magnitude larger than fluviatile inputs at the upstream boundaries (2001–2010: 0.15 10^6^ m^3^ yr^−1^) and net export rates from the lower Sea Scheldt to the outer estuary (2001–2010: 0.25 10^6^ m^3^ yr^−1^)^27^. This active transport of dredged material is a counter-gradient transport: all important dredging locations lie in the downstream part of MTZ1, while the dumping location lies in the upstream part (Fig. 1). Continuous addition of freshly dredged fine sediments at the dumping site results in easily erodable layer, part of which will thus be eroded and deposited again on the location where it was first dredged. Thus, current dredging and dumping activities in the Scheldt increase the residence time of fine sediments in MTZ1, hence we call this the *dredging pump*. No drastic increase in this dredging pump coinciding with the increasing SPM concentration can be observed in 2008; the total amount of relocated mud rather shows a consistent increase (Fig. 5B). The artificially enhanced residence could, however, have exceeded a critical threshold, above which sediments start to accumulate in MTZ1. This offers a second possible explanation for the observed transitions.

### The impact of water quality on sediment transport

One of the most intriguing findings is the gradual change in regression coefficients of SPM vs. freshwater discharge, coinciding with water quality improvement (Fig. 4). It is no surprise that changes in water quality can change properties of sediments^21^, but to our knowledge this is the first direct observation suggesting large scale impact in a natural system. This entails a potentially strong link between changes in eutrophication status and the distribution of sediments. There are multiple possible mechanisms to qualitatively explain the observations. First, note that in MTZ2a and MTZ2b, the tidal prism is larger in summer than in winter. Consequently shear rates can be expected to by higher in summer which would lead to larger resuspension rates and resulting SPM concentrations. The magnitude of this seasonality would depend on sediment erodability. Thus, an increasing erodability of sediments could potentially explain the increasing sensitivity of SPM towards freshwater discharge. An increase in sediment erodability in response to water quality improvement can have multiple causes. First, the decreasing ammonia and increasing oxygen concentrations are a proxy for decreasing (anthropogenic) organic matter (OM) inputs. It is reasonable to expect that decreasing OM inputs have led to decreasing OM content of the river bed. As OM content is a critical factor in the erodability of sediments^21^, a decrease in OM inputs would almost certainly lead to increasing erodability. However, the picture is more complex, as there are many more biota-mediated factors in sediment erodability. The stabilizing impact of biofilms of microphytobenthos is well-known^35,36^, as is the subsequent destabilizing impact of grazing by macrobenthos^37^. It is certain that total abundance and community structure of all sediment related biota have changed enormously with the changing water quality of the Scheldt. The question whether sediment erodability has indeed increased, and whether these changes might be due to water quality changes is complex and requires further study.

In addition, changes in water quality may have impacted flocculation of SPM in the water column. Flocs in natural waters constitute a complex matrix of microbial communities, organic and inorganic particles^38^. Thus, while SPM concentration and local turbulence are prime determinants of floc size^39,40^, also the organic matter and the microbial organisms that are associated with the flocs determine their aggregation and disaggregation rate, their strength and shape^22,41–43^. Particularly in the Scheldt, the decreasing OM and nutrient inputs, decreasing ammonium concentrations and increasing oxygenation, have been accompanied by a change in the balance between bacterial heterotrophic and chemo-autrophic metabolism (i.c. nitrification) on the one hand and photo-autotrophic metabolism by phytoplankton on the other^26^. Inevitably, these changes had an impact on the amount and type of organic matter and micro-organisms associated with flocs. The species composition of the microbial community can significantly impact floc structure e.g. by the way colonies are formed^44^. Also, the type and amount of extracellular polymeric substances (EPS) produced during the metabolism of bacteria and phytoplankton, depend on the species community and the substrate they grow on, their growth phases and stressors^45^. EPSs are generally believed to increase aggregation efficiency and enhance floc strength^22,46^. Combined, we can reasonably expect that biofeedbacks on flocculation dynamics have changed in response to water quality improvement efforts.

If anything, such a change in biofeedbacks would impact the seasonal variability of floc sizes. Both in the North Sea and in the Westerschelde such seasonality linked to (micro-) biological activity has been documented, with typically higher floc sizes in summer and smaller sizes in winter^47–49^. A seasonality in flocculation dynamics provides a potential mechanism for the existence of alternative sediment distribution patterns after 2008/2009. A study of MTZ location in the Ems to SPM settling velocity, Chernetsky *et al*. showed that the location and intensity of the MTZ depends on the settling velocity^15^. Their model showed a bifurcation in the MTZ location with increasing settling velocity (Fig. 17 in^15^). At low settling velocities, a single convergence zone was always found. At higher settling velocities, two MTZ locations were possible. The dynamics of the MTZ will thus be partly driven by the seasonality in settling velocities of natural aggregates, when they are lower during winter and higher during Spring and Summer. If settling velocities would periodically exceed the bifurcation threshold, it indeed depend on the exact trajectory of discharges, settling velocities, etc. whether SPM will end up in the upstream convergence zone or not.

## Conclusions

The SPM dynamics in the Scheldt estuary exhibited long term, gradual changes in the response to freshwater discharge, as well as abrupt changes in total amount of SPM and in its longitudinal distribution. The qualitative change from a single, weakly defined maximum turbidity zone (MTZ2a) with minor differences between summer and winter concentrations, to a clearly defined maximum turbidity zone that changes position in response to freshwater discharge (Fig. 2), represent the first direct observation of threshold behavior in estuarine SPM dynamics. Since 2009 the longitudinal distribution of SPM exhibits flickering behaviour between two co-existing alternative distributions, a behavior that is often seen before critical transitions, and may indicate that the system is vulnerable for a critical change^50,51^. We found that WQ improvement provides a strong potential explanation for changes in the SPM-Q relation. However, the mechanisms behind bioflocculation and biota-mediated sediment erodability are diverse and far from fully understood. Their relevance is increasingly recognized, but their relative importance remains largely unknown. Our observations support the hypothesis that they had large scale impacts on sediment distribution. Good mechanistic models, rooted in fundamental knowledge on the impacts of biogeochemical and biological processes on sediment erodability and aggregation dynamics are needed to further investigate this, but they are currently lacking.

## Material and Methods

### Sampling

Monthly or bi-weekly SPM data for 1996–2016 was retrieved from 2 environmental monitoring databases: the OMES database for the Belgian part of the estuary and the NIOZ database for the Dutch part. Sampling cruises, protocols and methods of the monitoring campaigns behind the databases were highly coordinated. Sampling cruises were tide independent, with no bias of sampling times in the timing with respect to the tidal cycle, nor with respect to the spring-neap cycle^52^. Samples were taken at 29 monitoring locations spread along the estuary (Fig. 1). Bucket samples were taken from aboard a ship during monthly and bi-weekly cruises. SPM was determined gravimetrically after filtration over pre-combusted GF/F filters. Other water quality parameters were determined using standard methods. Freshwater discharge data was provided by the Hydraulics Information Center of the Flanders Hydraulics Research.

### Data processing

#### Characterizing longitudinal SPM distribution

Due to continuous redistribution of SPM over the vertical in which transport processes at various timescale play a role, SPM data shows large variability. We used different averaging procedures to highlight different aspects of the data. To delineate different characteristic zones with respect to SPM concentrations, to identify two alternative SPM distribution patterns and for a first characterization of inter-annual variability, we averaged SPM data over the winter (Dec–May) and summer (Jun–Nov) half-years. To avoid bias to summer SPM concentrations, determined during bi-weekly cruises, data was month-averaged before averaging over 6 months.

### Breakpoint analysis

To pinpoint the timing and character of the change in dynamics of the different maximum turbidity zones, we constructed monthly time series by spatial averaging over the previously delineated zones. We used the change point model (CPM) framework to detect mean change in data sequences^53,54^. We tested the time series for change in mean of the log-transformed time series using the standard Student t-test. The Student t-test assumes a Gaussian distribution. The normality of the log-transformed subsets before and after the detected breakpoint was confirmed a posteriori, with a Shapiro-Wilk test for all subsections except the post 2009 section in MTZ2a (MTZ1, t < 2008: p = 0.49, n = 142; MTZ1, t >= 2008: p = 0.22, n = 120; MTZ2b, t < 2009: p = 0.4, n = 99; MTZ2b, t >= 2009: p = 0.9, n = 104; MTZ2a, t < 2009: p = 0.29, n = 153; MTZ2a, t >= 2009: p = 0.002, n = 108). Normality was further visually inspected with Quantile plots (Supplementary Fig. 1). Since only weak autocorrelation exists in the monthly time series (Supplementary Fig. 2) a standard t-test can be used to for change in mean^55^. Differences in mean value of log-transformed monthly time series before and after the detected breakpoints were significant in MTZ1 (df = 230, p < 1e-13) and MTZ2b (df = 131, p = 0.0013). In MTZ2b, after 2009 some years have elevated SPM concentrations, while others have SPM concentrations similar to the period before 2009. The difference between years with elevated concentrations and years prior to 2009 grouped with low concentration years was significant (df = 76, p = 0.0005).

### Lagged regressions

To characterize the relation of the two freshwater maximum turbidity zones with freshwater discharge (Q) on a seasonal time scale, we used central moving averages of 6 month width to separate longer term trends from short term variability in the SPM and Q time series. Subsequently we performed lagged linear regressions in moving windows of 1 year width on these smoothed time series, by total least squares minimization. In each window (252 in MTZ2a, and 198 in MTZ2b) we performed lagged regressions within a predefined lag-range. Among the lagged regressions we selected the lag resulting in the lowest sum of squared residuals (SSR); if 2 minima were present within the lag range, we selected the lag closest to the overall average lag (this was the case for 8 and 9 regressions in MTZ2a and MTZ2b respectively). We used SSR instead of R^2^ as a robust measure, also for regressions with low or zero slope. Because of the seasonality in both Q and SPM, a positive correlation at lag *j* [months] is accompanied by a negative correlation at lag *j ± 6*, and vice versa. Since random effects could result in a lower SSR at unphysical larger lags, we avoided such spurious lag selection by imposing a 6 month difference between maximal and minimal lag. These were iteratively centered around the average selected lag over the whole period. Thus, for MTZ2a lagged regressions with a lag between between −2 and +4 [months] was performed; for MTZ2b lags were varied between −3 and +3. The large majority of regressions had a clear minimum in the SSR within the predefined lag range (Supplementary Fig. 3). Only 11 and 3 regressions had minimal SSR at the largest lags in the imposed range, further justifying the predefined lag ranges. The SSR was also used to eliminate regressions with low explanatory power; specifically we ran an outlier detection algorithm on the time series of SSR of selected regressions. SSRs higher than the average SSR + 2.5 standard deviations were identified as an outlier. This was performed iteratively until no further outliers were detected (Supplementary Fig. 4).

## Supplementary information

Supplementary material

## Data Availability

The data of the Belgian Scheldt can be obtained for scientific purposes by simple request to De Vlaamse Waterweg, owner of this data. The data of the Westernscheldt is available for download on www.rforscience.com.

## References

[1] Burchard, H., Schuttelaars, H. M. & Ralston, D. K. Sediment Trapping in Estuaries, *in* Carlson, C. A. & Giovannoni, S. J., eds, ‘Annual Review Of Marine Science, Vol 10’, *Annual Reviews*, pp. 371–395 (2018). 10.1146/annurev-marine-010816-060535 28977760

[2] Li P (2012). Spatial, Temporal, and Human-Induced Variations in Suspended Sediment Concentration in the Surface Waters of the Yangtze Estuary and Adjacent Coastal Areas. Estuaries and Coasts.

[3] Liu JH, Yang SL, Zhu Q, Zhang J (2014). Controls on suspended sediment concentration profiles in the shallow and turbid Yangtze Estuary. Continental Shelf Research.

[4] Hoa LTV, Nguyen HN, Wolanski E, Tran TC, Haruyama S (2007). The combined impact on the flooding in Vietnam’s Mekong River delta of local man-made structures, sea level rise, and dams upstream in the river catchment. Estuarine, Coastal and Shelf Science.

[5] Lesourd S (2001). Morphosedimentary evolution of the macrotidal Seine estuary subjected to human impact. Estuaries.

[6] Le Hir P (2001). Fine sediment transport and accumulations at the mouth of the Seine estuary (France). Estuaries.

[7] Collins MJ, Miller D (2012). Upper Hudson river estuary (USA) Floodplain change of the 20th century. River research and applications.

[8] Jalón-Rojas I, Schmidt S, Sottolichio A (2015). Turbidity in the fluvial Gironde Estuary (southwest France) based on 10-year continuous monitoring: sensitivity to hydrological conditions. Hydrology and Earth System Sciences.

[9] Kerner M (2007). Effects of deepening the Elbe Estuary on sediment regime and water quality. Estuarine Coastal And Shelf Science.

[10] Geyer WR, Woodruff JD, Traykovski P (2001). Sediment transport and trapping in the Hudson River estuary. Estuaries.

[11] Jalón-Rojas I, Schmidt S, Sottolichio A (2017). Comparison of environmental forcings affecting suspended sediments variability in two macrotidal, highly-turbid estuaries. Estuarine, Coastal and Shelf Science.

[12] de Jonge VN, Schuttelaars HM, van Beusekom JEE, Talke SA, de Swart HE (2014). The influence of channel deepening on estuarine turbidity levels and dynamics, as exemplified by the Ems estuary. Estuarine Coastal And Shelf Science.

[13] Winterwerp JC, Wang ZB (2013). Man-induced regime shifts in small estuaries-I: theory. Ocean Dynamics.

[14] Winterwerp JC, Wang ZB, van Braeckel A, van Holland G, Koesters F (2013). Man-induced regime shifts in small estuaries-II: a comparison of rivers. Ocean Dynamics.

[15] Chernetsky AS, Schuttelaars HM, Talke SA (2010). The effect of tidal asymmetry and temporal settling lag on sediment trapping in tidal estuaries. Ocean Dynamics.

[16] Donker JJA, de Swart HE (2013). Effects of bottom slope, flocculation and hindered settling on the coupled dynamics of currents and suspended sediment in highly turbid estuaries, a simple model. Ocean Dynamics.

[17] Winterwerp JC (2011). Fine sediment transport by tidal asymmetry in the high-concentrated Ems River: indications for a regime shift in response to channel deepening. Ocean Dynamics.

[18] Toorman, E., Bruens, A., Kranenburg, C. & Winterwerp, J. Interaction of suspended cohesive sediment and turbulence. *In* Winterwerp, J. C. & Kranenburg, C., eds, ‘fine Sediment Dynamics In The Marine Environment’,. 6th International Conference on Cohesive Sediment Transport (INTERCOH 2000), Delft, Netherlands, Sep 04-08, 2000. *Proceedings in Marine Science* **5***. 7–23* (2002),

[19] Winterwerp JC, Lely M, He Q (2009). Sediment-induced buoyancy destruction and drag reduction in estuaries. Ocean Dynamics.

[20] Dijkstra YM, Schuttelaars HMWJC (2018). The hyperturbid state of the water column in estuaries and rivers: the importance of hindered settling. Ocean Dynamics.

[21] Grabowski R, Droppo I, Wharton G (2011). Erodibility of cohesive sediment: The importance of sediment properties. Earth-Science Reviews.

[22] Lai H, Fang H, Huang L, He G, Reible D (2018). A review on sediment bioflocculation: Dynamics, influencing factors and modeling. Science of The Total Environment.

[23] Talke S, de Swart H, Schuttelaars H (2009). Feedback between residual circulations and sediment distribution in highly turbid estuaries: An analytical model. Continental Shelf Research.

[24] Meire P (2005). The Scheldt estuary: a description of a changing ecosystem. Hydrobiologia.

[25] Soetaert K (2006). Long-term change in dissolved inorganic nutrients in the heterotrophic Scheldt estuary (Belgium, The Netherlands). Limnology and Oceanography.

[26] Cox TJS (2009). A macro-tidal freshwater ecosystem recovering from hypereutrophication: the Schelde case study. Biogeosciences.

[27] Vandenbruwaene, W. *et al*. Integraal plan Boven‐Zeeschelde: Deelrapport 8 – Sedimentbalans Zeeschelde, Rupel en Durme. Versie 4.0. WL Rapporten, 13_131_8. Waterbouwkundig Laboratorium: Antwerpen (2017).

[28] Mialet B (2011). Response of zooplankton to improving water quality in the Scheldt estuary (Belgium). Estuarine Coastal and Shelf Science.

[29] Chambord S (2016). Mesozooplankton affinities in a recovering freshwater estuary. Estuarine Coastal and Shelf Science.

[30] Vandaele K, Poesen J (1995). Spatial And Temporal Patterns Of Soil-erosion Rates In An Agricultural Catchment, Central Belgium. Catena.

[31] Steegen A (2000). Sediment export by water from an agricultural catchment in the Loam Belt of central Belgium. Geomorphology.

[32] Allen G, Salomon J, Bassoullet P, Penhoat YD, de Grandpré C (1980). Effects of tides on mixing and suspended sediment transport in macrotidal estuaries. Sedimentary Geology.

[33] Savenije HH (1993). Composition and driving mechanisms of longitudinal tidal average salinity dispersion in estuaries. Journal of Hydrology.

[34] Kuijper, K. & Lescinski, J. LTV Veiligheid & Toegankelijkheid Data analyses water levels ebb and flood volumes and bathymetries Western Scheldt. Deltares (2013).

[35] Gerbersdorf SU, Wieprecht S (2015). Biostabilization of cohesive sediments: revisiting the role of abiotic conditions, physiology and diversity of microbes, polymeric secretion, and biofilm architecture. Geobiology.

[36] de Brouwer J, Wolfstein K, Ruddy G, Jones T, Stal L (2005). Biogenic stabilization of intertidal sediments: The importance of extracellular polymeric substances produced by benthic diatoms. Microbial Ecology.

[37] de Deckere E, Tolhurst T, de Brouwer J (2001). Destabilization of cohesive intertidal sediments by infauna. Estuarine Coastal and Shelf Science.

[38] Droppo I (2001). Rethinking what constitutes suspended sediment. Hydrological Processes.

[39] Dyer K (1989). Sediment processes in estuaries – Future research requirements. Journal of Geophysical Research-Oceans.

[40] Pejrup M, Mikkelsen OA (2010). Factors controlling the field settling velocity of cohesive sediment in estuaries. Estuarine Coastal and Shelf Science.

[41] Maggi F (2009). Biological flocculation of suspended particles in nutrient-rich aqueous ecosystems. Journal of Hydrology.

[42] Maggi F, Tang FH (2015). Analysis of the effect of organic matter content on the architecture and sinking of sediment aggregates. Marine Geology.

[43] Mietta F, Chassagne C, Manning AJ, Winterwerp JC (2009). Influence of shear rate, organic matter content, pH and salinity on mud flocculation. Ocean Dynamics.

[44] de Lucas Pardo MA, Sarpe D, Winterwerp JC (2015). Effect of algae on flocculation of suspended bed sediments in a large shallow lake. Consequences for ecology and sediment transport processes. Ocean Dynamics.

[45] More TT, Yadav JSS, Yan S, Tyagi RD, Surampalli RY (2014). Extracellular polymeric substances of bacteria and their potential environmental applications. Journal of Environmental Management.

[46] Fettweis M, Baeye M, Van der Zande D, Van den Eynde D, Lee BJ (2014). Seasonality of floc strength in the southern North Sea. Journal of Geophysical Research-Oceans.

[47] Fettweis M, Baeye M (2015). Seasonal variation in concentration, size, and settling velocity of muddy marine flocs in the benthic boundary layer. Journal of Geophysical Research-Oceans.

[48] van der Wal D, van Kessel T, Eleveld MA, Vanlede J (2010). Spatial heterogeneity in estuarine mud dynamics. Ocean Dynamics.

[49] Shen X, Toorman EA, Lee BJ, Fettweis M (2018). Biophysical flocculation of suspended particulate matters in Belgian coastal zones. Journal of Hydrology.

[50] Dakos V, van Nes EH, Scheffer M (2013). Flickering as an early warning signal *Theoretical*. Ecology.

[51] Scheffer M (2009). Early-warning signals for critical transitions. Nature.

[52] Vandenbruwaene, W., Vanlede, J., Plancke, Y., Verwaest, T., & Mostaert, F. Slibbalans Zeeschelde: deelrapport 4. Historische evolutie SPM (2016).

[53] Hawkins D, Qiu P, Kang C (2003). The changepoint model for statistical process control. Journal Of Quality Technology.

[54] Ross G (2015). J. arametric and nonparametric sequential change detection in R: The cpm package. Journal of Statistical Software.

[55] Zwiers FW, von Storch H (1995). Taking Serial Correlation into Account in Tests of the Mean. Journal of Climate.

