# Supplementary material for: Critical transitions in suspended sediment dynamics in a temperate meso-tidal estuary
Source: Sci Rep. 2019 Sep 4;9:12745. doi: 10.1038/s41598-019-48978-5 (PMC6726847; doi:10.1038/s41598-019-48978-5)
Supplement: Supplementary file 1 — Supplementary material [file 41598_2019_48978_MOESM1_ESM.pdf]

Supplementary material

Critical transitions in suspended sediment dynamics in a temperate meso-tidal estuary.

Cox, T.J.S.[1,2,*] (tom.cox@uantwerpen.be)

Maris, T.[1]

Van Engeland, T.[1]

Soetaert, K.[2]

Meire, P.[1]

1. Ecosystem Management Research Group, University of Antwerp. Universiteitsplein 1C, 2610 Wilrijk (Belgium).

2. Estuarine and Delta Systems, Netherlands Institute of Sea Research (NIOZ). P.O. Box 140, 4400 AC Yerseke (The Netherlands).

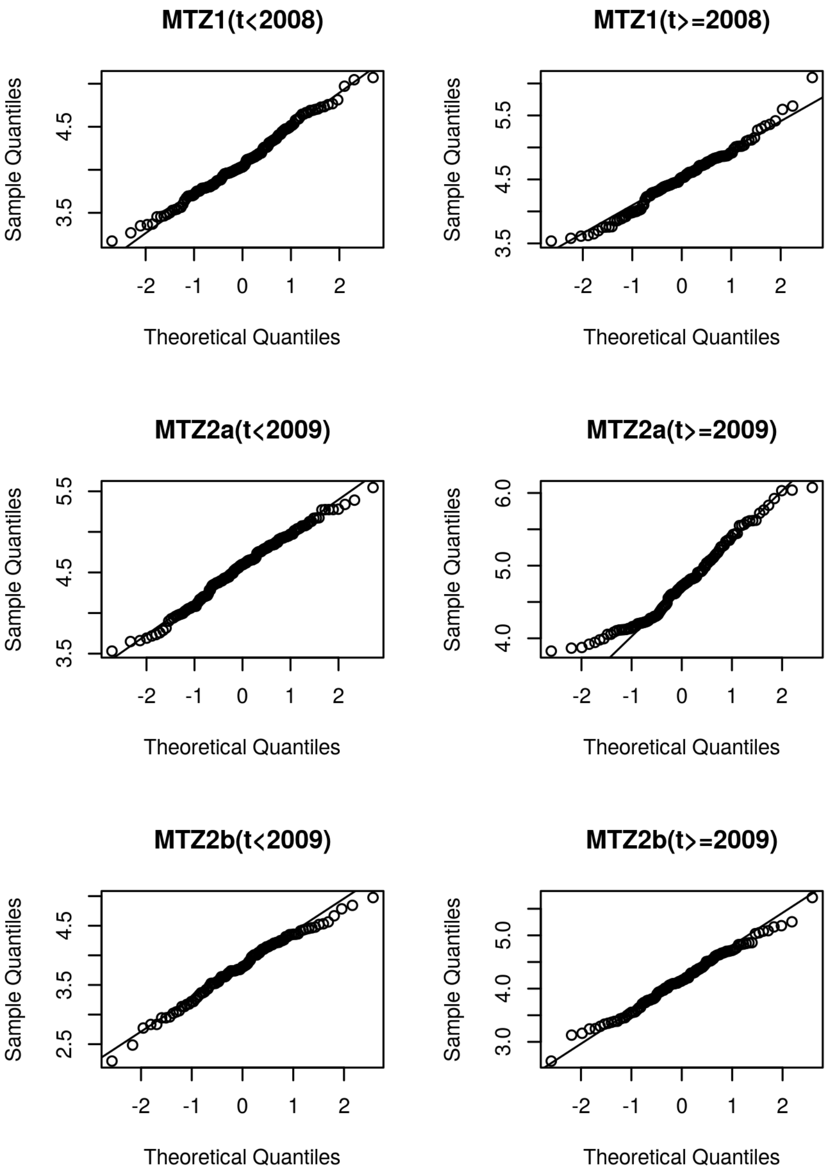

Supplementary Figure 1. Quantile plots for visual inspection of normality of log-transformed monthly SPM time series in the different MTZs, before and after breakpoints.

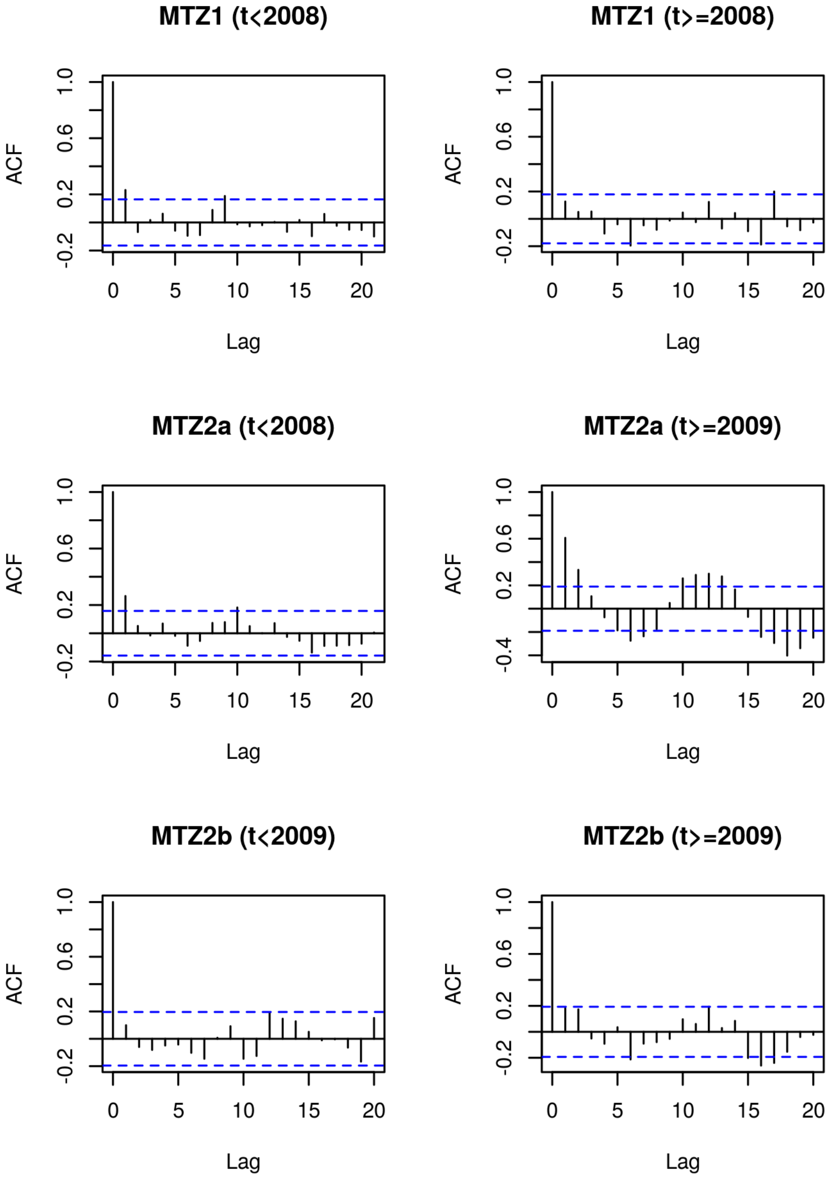

Supplementary Figure 2. Sample autocorrelation function of log-transformed monthly SPM time series in the different MTZs, before and after breakpoints

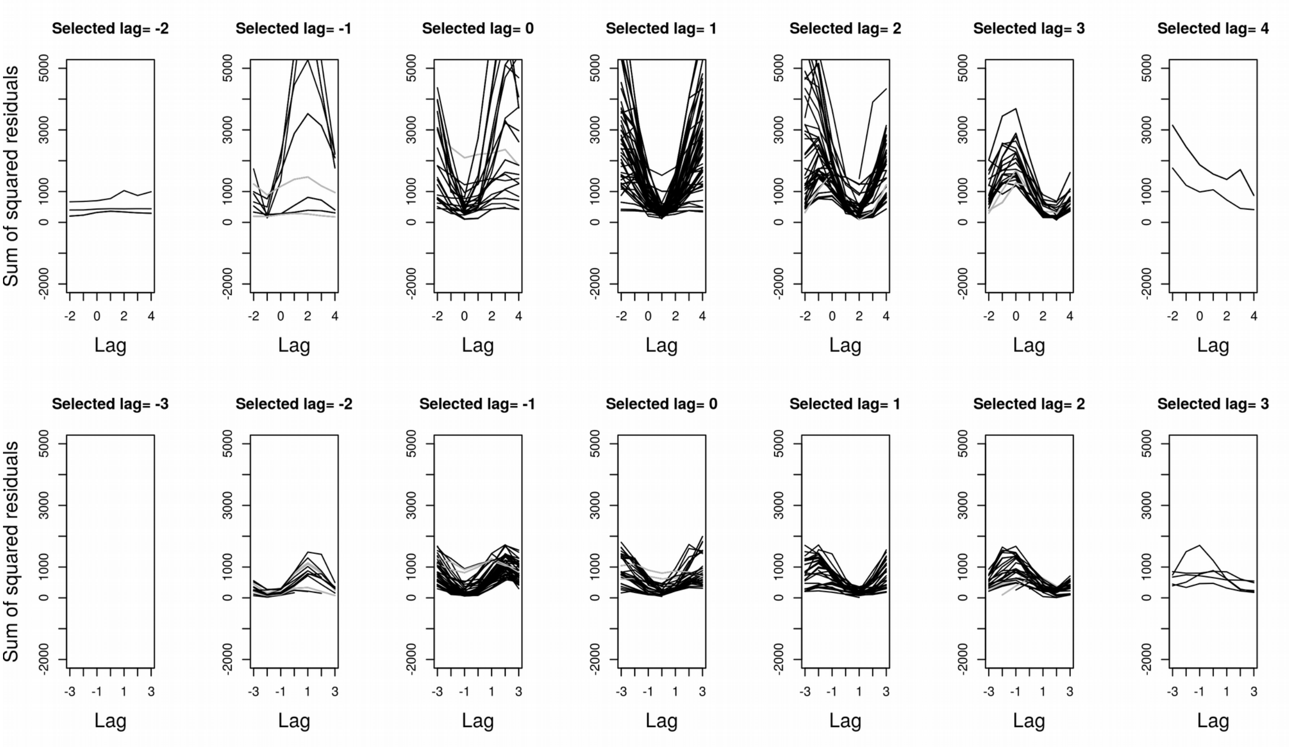

Supplementary Figure 3 Selection of lag with minimal Sum of squared residuals. Each line represents linear orthogonal regressions in a 12 month window, obtained at all lags in a 6 month range. Subsequently, the lag resulting in the lowest sum of squared residuals was selected in each window.

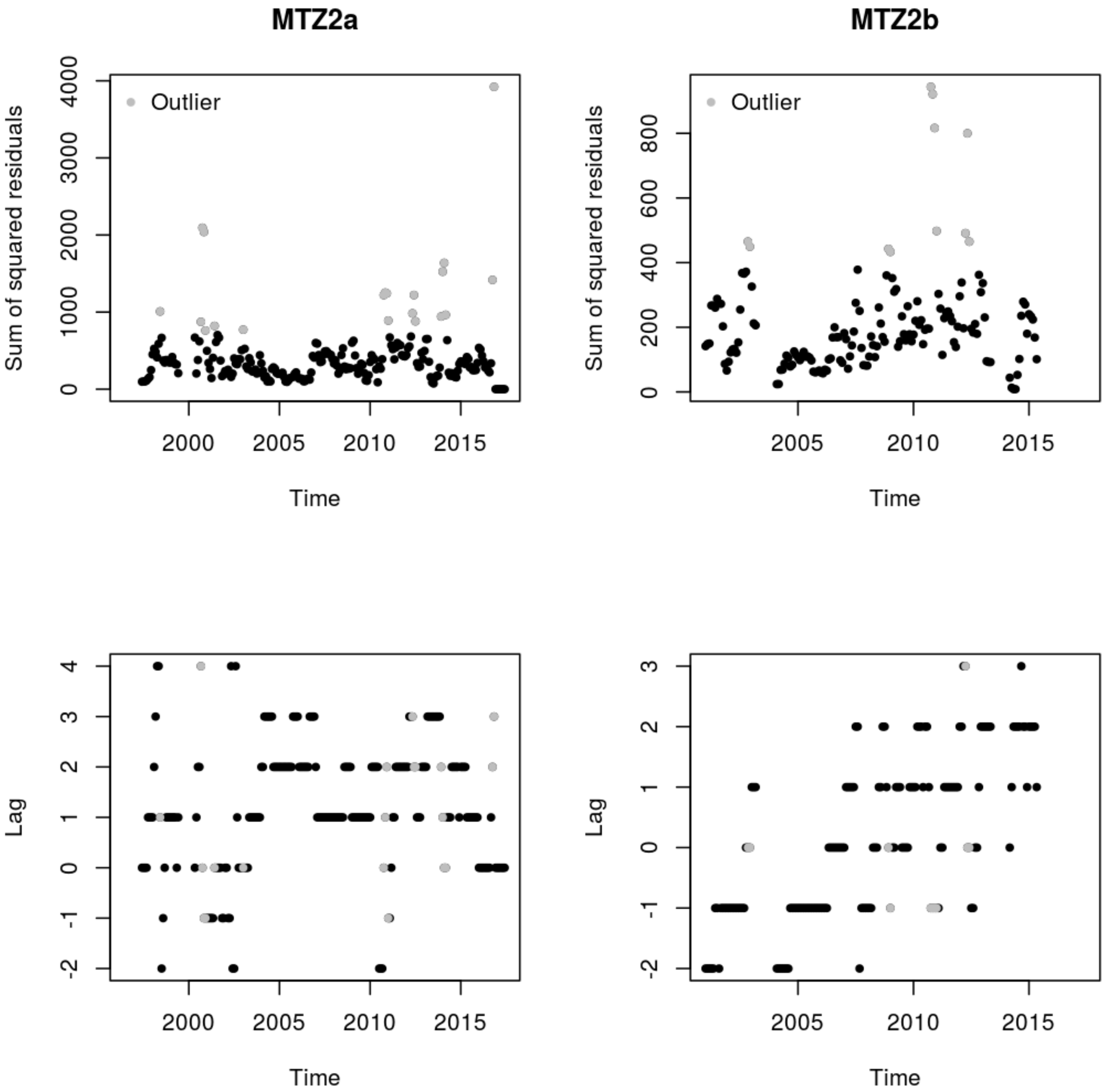

Supplementary Figure 4 Outlier detection in sum of squared residuals (top). Lags corresponding to minimal sum of squared residuals (bottom).
